# Flexible Foraging Strategies of Hainan Gibbons ( *Nomascus hainanus* ) in Response to Food Variation Across Contrasting Habitats

**DOI:** 10.1002/ece3.73703

**Published:** 2026-05-21

**Authors:** Wei Yang, Dexu Zhang, Shuai Liu, Kening Lu, Chuyue Yu, Changyuan Su, Yuan Chen, Wenxing Long

**Affiliations:** ^1^ School of Tropical Agriculture and Forestry Hainan University Danzhou China; ^2^ Collaborative Innovation Center of Ecological Civilization Hainan University Haikou China; ^3^ The National Conservation and Research Centre of Gibbon Changjiang China; ^4^ The Conservation and Research Centre of Hainan Gibbon Hainan University Haikou China; ^5^ School of Ecology Hainan University Haikou China

**Keywords:** composite correlated random walk, foraging movement, resource variation, small ape

## Abstract

Animals exhibit different foraging strategies in environments characterized by patchily distributed resources and seasonal variation. Hainan gibbons ( *Nomascus hainanus* ) are Critically Endangered primates with a largely frugivorous diet. They inhabit tropical rainforests where food resources are characterized by pronounced spatiotemporal heterogeneity; however, their foraging strategies remain poorly understood. We conducted field monitoring on two groups of Hainan gibbons from March 2023 to August 2025 in the Bawangling region of Hainan Tropical Rainforest National Park, China. Group C inhabited higher‐altitude montane rainforest, whereas Group E inhabited disturbed secondary lowland rainforest. Gibbon behaviors were recorded at 5‐min intervals using instantaneous scan sampling, and the spatial locations of behaviors were documented at 15‐min intervals. The results showed that, at the spatial scale, Group C exhibited higher movement directionality and spent a greater proportion of time in the extensive search phase (pE = 0.22) than Group E (pE = 0.04); this phase was characterized by straighter, long‐distance movements. In contrast, Group E spent a lower proportion of time in extensive search and exhibited longer mean step lengths but weaker directionality. At the temporal scale, gibbons primarily engaged in short‐distance, localized intensive search during the mixed‐diet period (October–March, characterized by lower fruit availability), whereas they spent more time in the extensive search phase during the fruit‐eating phase (April–September, when fruit resources were more abundant). Overall, Hainan gibbons flexibly switched between extensive and intensive search modes to achieve directional inter‐patch movements while maximizing within‐patch exploitation, thereby effectively coping with temporal and spatial variation in fruit resources. These findings indicate that movement behavior in Hainan gibbons is closely associated with habitat characteristics and the spatial and temporal distribution of food resources. Our study also highlights that low‐altitude tropical secondary forests are important for the survival of the Hainan gibbons and thus have high conservation value.

## Graphical abstract

This study, based on long‐term field monitoring and conservation efforts of Hainan gibbons, analyzed their foraging movement strategies across different spatial contexts (Groups C and E) and temporal periods (the fruit‐eating period and the mixed‐diet period). Overall, Hainan gibbons flexibly switch between extensive and intensive search modes to achieve directional inter‐patch movements while maximizing within‐patch exploitation, thereby effectively coping with temporal and spatial variation in fruit resources. These findings provide important insights for habitat management and conservation planning for Hainan gibbons in heterogeneous environments.

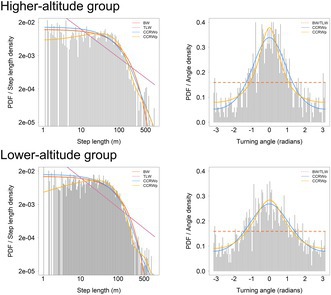

## 1 Introduction

Animal movement represents a fundamental ecological process through which individuals acquire energy, reproduce, and persist in complex environments (Nathan et al. 2008; Holyoak et al. 2008). This movement is driven by the interplay between internal states, including motivation, locomotor capacity, navigational ability, and external environmental conditions (Nathan et al. 2008). Consequently, movement is a critical mechanism for coping with environmental change, as it directly determines the capacity of an individual to acquire energy (Goossens et al. 2020; Abrahms et al. 2021). The efficiency of this search behavior fundamentally shapes foraging patterns and fitness outcomes (Pyke 1984; Williams and Safi 2021). By adjusting their search strategies, animals can increase encounter rates with resources in dynamic environments (Zollner and Lima 1999), thereby balancing movement costs against energetic gains to maximize net energy acquisition.

Understanding how animals move in dynamic environments is fundamental to elucidating their strategies for resource use. Movement strategies are highly flexible and context dependent, with individuals adjusting their tactics in response to variation in resource density and predictability (Charnov 1976; Abrahms et al. 2021). Animal movement patterns are not fixed, but instead represent behavioral responses to the spatiotemporal distribution of external food resources. Variation in resource density, dispersion, and predictability influences the distributions of step lengths and turning angles, thereby shaping different random walk movement patterns (e.g., Brownian Walk (BW), Lévy Walk (LW), or Composite Correlated Random Walk (CCRW)) (López‐López et al. 2013; Vidal‐Mateo et al. 2022).

Among the analytical frameworks developed to quantify foraging behavior, random walk models provide a theoretical basis for describing individual movement trajectories (Codling et al. 2008). Classical Brownian movement is characterized by exponentially distributed step lengths and random turning angles, making it suitable for environments with homogeneously distributed resources (Codling et al. 2008; Reyna‐Hurtado et al. 2018). In contrast, the LW model features step lengths that follow a power law distribution, with many short steps interspersed with occasional long relocations, a strategy efficient for increasing resource encounters in sparse, patchy environments (Ramos‐Fernández et al., 2004; López‐López et al. 2013). Early theoretical work proposed the Lévy walk as an optimal search strategy for locating sparsely and randomly distributed resources, particularly under conditions of low resource density and high unpredictability (Viswanathan et al. 1996). Empirical evidence from species such as jackals ( *Lycaon pictus* ) (Atkinson et al. 2002), Cory's shearwater (*Calonectris borealis*), Yelkouan shearwater ( *Puffinus yelkouan* ) (Reynolds et al. 2016), Gray‐cheeked mangabey ( *Lophocebus albigena* ) and spider monkey ( *Ateles geoffroyi yucatanensis* ) (Reyna‐Hurtado et al. 2018) further demonstrated that animal movement often approximates a power law distribution, characterized by a heavy‐tailed distribution that allows for occasional long‐distance displacements when resources are highly irregular. CCRW describes a search strategy composed of two behavioral modes—the extensive search and the intensive search—which, compared with single‐mode movement, confers advantages in heterogeneous landscapes (Auger‐Méthé et al. 2015, 2016). The extensive search phase involves rapid, directed movement between resource patches, while the intensive search phase is characterized by slower speeds and more sinuous paths within patches (Auger‐Méthé et al. 2016). By switching between these two behavioral modes, animals can adjust their movement according to patch density and distribution, enabling longer residence times in resource‐rich areas even when patch boundaries are indistinct (Benhamou and Collet 2015). The CCRW can be used to distinguish between localized foraging and broad scale movement phases and can quantitatively assess how resource variation drives changes in search strategies (Auger‐Méthé et al. 2016; Leos‐Barajas et al. 2017).

In primates and other animals with advanced cognitive abilities, movement strategies are closely shaped by the spatial and temporal distribution of food resources. Research shows that primates employ diverse movement patterns during spatial exploration. For example, spider monkeys ( *Ateles geoffroyi* ), chacma baboons ( *Papio ursinus* ), and Tonkean macaques ( *Macaca tonkeana* ) exhibit step length and residence time distributions consistent with either Lévy or Brownian movement patterns, in response to food resources that are patchily distributed in space and highly variable across seasons (Ayala‐Orozco et al. 2004; Boyer et al. 2006; Sueur 2011; Gursky‐Doyen et al. 2011). Furthermore, some species exhibit mixed‐movement characteristics. For instance, the bearded saki ( *Chiropotes sagulatus* ) employs a mixed strategy: step lengths within resource‐dense patches approximate Brownian motion, whereas movements between heterogeneous patches follow a power law distribution, reflecting long‐distance exploration between patches (Shaffer 2014). In summary, primate foraging strategies are primarily shaped by the spatial distribution of resources. However, the fine‐scale adjustment of these strategies within complex tropical forest habitats remains poorly understood.

Hainan gibbon ( *Nomascus hainanus* ) is one of the world's most Critically Endangered primates (IUCN 2020), with a global population of only seven groups (42 individuals) confined to approximately 15 km^2^ of tropical rainforest in the Bawangling region of Hainan Tropical Rainforest National Park (Turvey et al. 2024; Zhong et al. 2025). Historical human disturbances, including large‐scale forest destruction and conversion of low‐elevation rainforest into pine plantations, have contributed to environmental differences among the habitats occupied by Hainan gibbon groups (Liu et al. 2023; Lu et al. 2025). Group C inhabits a tropical montane rainforest at elevations ranging from 613 to 1021 m, characterized by high plant diversity and abundant large fruiting trees, resulting in relatively stable fruit availability (Zhang et al. 2022; Wang et al. 2025; Du et al. 2020). In contrast, Group E inhabits a secondary tropical lowland rainforest between 571 and 872 m with a simplified structure and substantial human disturbance; previous studies indicate that large fruiting trees are fewer, and fruit availability is lower and more variable across space and time (Zhang et al. 2022; Wang et al. 2025; Du et al. 2020). Based on these habitat differences, we predicted that the two groups would exhibit different movement patterns. Specifically, we expected Group C, which occupies a habitat with relatively higher and more stable fruit availability, to show more directional movement and more frequent travel between food patches. In contrast, we expected Group E, which occupies a more disturbed and resource‐variable habitat, to show more localized and tortuous movement. We also predicted that movement patterns would vary across periods with different dietary composition, with relatively more directed inter‐patch movement during periods with a higher proportion of fruit in the diet and more localized movement during mixed‐diet periods. In this study, the movement strategies of Groups C and E were investigated using an integrated framework based on three movement models: LW, BW, and CCRW. This investigation will advance the understanding of the ecological adaptations of Hainan gibbons and provide scientific guidance for the habitat management and conservation of this Critically Endangered species.

## 2 Materials and Methods

### 2.1 Study Site

**FIGURE 1:**
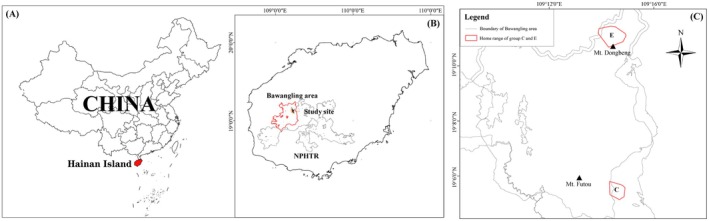
Maps of the study site. (A) Map of China showing the location of Hainan Island; (B) Map of Bawangling region in Hainan Island; (C) Distribution of the study area within the Bawangling. The letter C represents Group C (higher‐altitude group), while the letter E represents Group E (lower‐altitude group).

The study was conducted in the Bawangling region of Hainan Tropical Rainforest National Park (18°48′–19°12′N, 108°55′–109°17′E) (Figure 1). This area experiences a tropical monsoon climate, with a mean annual temperature of 23.6°C and an average annual precipitation of 1677.1 mm (Long et al. 2011; Li et al. 2023). The climate is characterized by distinct dry (November–April) and rainy (May–October) seasons (Ding et al. 2017; Fan et al. 2021). The region has been subject to historical anthropogenic disturbances, with only approximately 4% (about 12 km^2^) of the original primary forest remaining (Ding et al. 2012). The current landscape is fragmented and dominated by secondary forests that have regenerated following these disturbances. The current population of the Hainan gibbon has seven groups totaling 42 individuals, with Groups C and E were selected as subjects. Group C (hereafter referred to as the higher‐altitude group) inhabits an old‐growth tropical montane rainforest characterized by relatively high habitat quality and abundant fruiting plants (Zhang et al. 2022; Liu et al. 2023). This group consists of nine individuals, including two adult males, two adult females, one subadult, two juveniles, and two infants. Group E (hereafter referred to as the lower‐altitude group) inhabits a secondary tropical lowland rainforest that previous studies have described as having relatively lower and more variable food resource availability (Zhang et al. 2024; Liu et al. 2025). This group consists of four individuals, including one adult male, one adult female, one juvenile, and one infant.

### 2.2 Data Collection

Each group was monitored for 10 days per month from March 2023 to August 2025. Before the formal study period, both groups had been regularly monitored for more than 1 year and were sufficiently habituated to human observers, allowing behavioral observations and GPS tracking with minimal disturbance. We employed instantaneous scan sampling at 5‐min intervals to record behavioral categories (traveling, feeding). Concurrently, group locations were recorded every 15 min using a handheld GPS unit. Data were collected daily from 06:00 to 18:00, until the gibbons reached their sleeping sites or the group was lost from view (e.g., in steep terrain). We defined a valid trajectory segment as a sequence of at least four consecutive GPS locations collected over a period of no less than 60 min. Step length was defined as the Euclidean distance between two successive locations, and turning angle was defined as the difference between two successive heading angles (see details below). Segments with fewer than four consecutive GPS locations were excluded from further analysis to maintain consistency in trajectory segmentation and to minimize potential biases associated with short or incomplete movement sequences. In total, we obtained 1593 valid GPS locations for higher‐altitude group and 2593 for lower‐altitude group.

### 2.3 Data Analysis

GPS locations were converted to a projected coordinate system (UTM), and outlier points were removed. Movement data were quantified by calculating step lengths and turning angles. Step length (*L*_*t*_, m) was defined as the straight‐line distance between two consecutive GPS points recorded at 15‐min intervals. The formula is as follows:

$$L_{t}=\sqrt{{\left(x_{t+1}-x_{t}\right)}^{2}+{\left(y_{t+1}-y_{t}\right)}^{2}}$$

The turning angle for each movement step (*φ*_*t*_) was defined as the directional angle from point (*x*_*t*_, *y*_*t*_) to the subsequent point (*x*_*t*+1_, *y*_*t*+1_). The formula is as follows:

$$\varphi_{t}=\text{atan}2\left(y_{t+1}-y_{t},x_{t+1}-x_{t}\right)$$

The turning angle (*θ*_*t*_, in radians) was defined as the difference between the heading directions of two consecutive steps. The formula is as follows:

$$\theta_{t}=\text{atan}2\left(\sin\left(\varphi_{t}-\varphi_{t-1}\right),\cos\left(\varphi_{t}-\varphi_{t-1}\right)\right),\theta_{t}\in \left[-\pi ,\pi \right]$$

Step length and turning angle calculations were implemented using the R package *trajr* (McLean and Skowron Volponi 2018).

**FIGURE 2:**
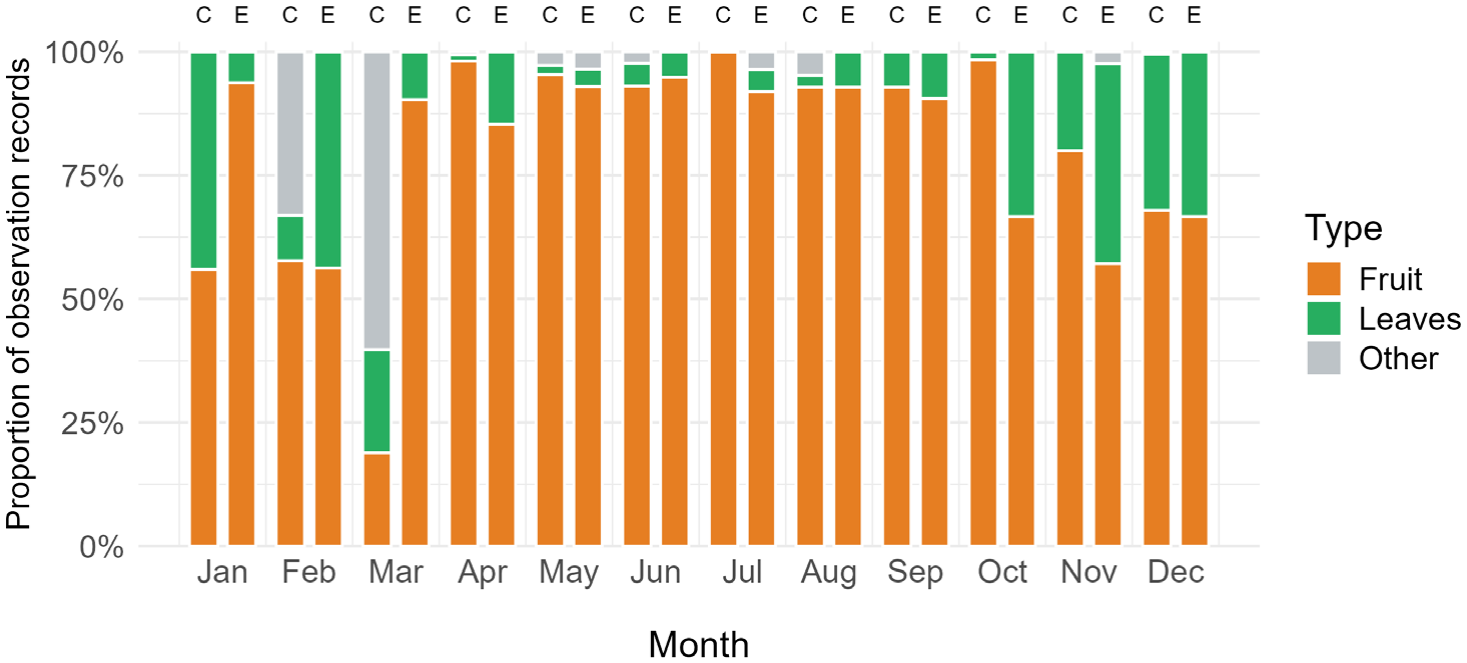
Proportion of different food items consumed by Hainan gibbons across different months. The letter C represents Group C (higher‐altitude group), while the letter E represents Group E (lower‐altitude group).

This study used long‐term monitoring data covering multiple annual cycles (March 2023–August 2025) to compare the movement patterns of Groups C and E. Hainan gibbons also show seasonal differences in food choice. Previous studies have shown that Hainan gibbons exhibit clear seasonal variation in food choice, with fruit contributing a higher proportion of the diet during the rainy season, whereas leaves, flowers, and insects contribute more during the dry season (Du et al. 2020; Zhong et al. 2023; Wang et al. 2025). To further examine differences in movement patterns under different food resource conditions, we divided the year into two feeding periods based on the proportions of different food types consumed by Hainan gibbons in the present study (Figure 2): (1) Fruit‐eating (April–September): During this period, fruiting food plants have a high level of fruit maturity, making it the main fruit feeding period for the gibbons. (2) Mixed‐period (October–March): Fruits are relatively scarce, and gibbons feed on a mixture of fruits, leaves, flowers, and insects. Using this classification, we compared gibbon movement patterns between the fruit‐eating period and the mixed‐diet period.

Referring to Auger‐Méthé et al. (2016), trajectory data were fitted and compared using four random walk models: BW, TLW, and CCRW. These models were applied to the step length and turning angle data of Hainan gibbons to identify the foraging search strategies adopted under different environmental conditions. We examined three representative search strategies and their corresponding biological meanings. LW search strategy represents the movement pattern in which animals employ long distance movements under conditions of sparse and highly patchy resources, and is expressed using the truncated TLW. Step lengths follow the truncated Pareto distribution (Truncated Pareto, w (l)), where w (l) denotes the probability density function defined over a finite interval with lower and upper bounds, allowing the representation of occasional long‐distance movements and capturing the behavioral characteristics of gibbons during long‐distance movements. The Brownian search strategy reflects random short distance movements when animals forage in relatively homogeneous or locally available resources. This strategy was represented by the BW model, in which step lengths are described by an exponential distribution (Exponential, φ(l)). The turning angle distributions of both the LW search strategy and the BW search strategy are expressed using the circular uniform distribution (Uniform, *v*₀ (h)). CCRW describes the switching between intensive search and extensive search movement modes, including two behavioral phases: an intensive phase (I), characterized by short step lengths and large turning angles, indicating localized search within a limited area; and an extensive phase (E), characterized by longer step lengths and stronger directional persistence, indicating movement between areas. We used two variants of the CCRW, the Hidden Markov model and the Hidden semi‐Markov model, which are expressed as CCRWo and CCRWp, respectively.

In the CCRWo model, step lengths follow the exponential distribution *φ* (l), turning angles in the intensive search phase follow the circular uniform distribution v₀ (h), and turning angles in the extensive search phase follow the von Mises distribution *v* (h) centered at 0, a circular distribution commonly used to model directional data with a preferred orientation. The residence time follows the exponential distribution *φ* (l), with residence times showing no clear tendency toward longer or shorter durations. In the CCRWp model, step lengths follow the Weibull distribution, a flexible distribution commonly used to describe movement distances with varying shapes. Turning angles follow the Wrapped Cauchy distribution, a circular distribution used to model directional data, where higher concentration indicates stronger directional persistence, with the turning angle concentration in the extensive search phase being greater than 0. The residence time was modeled using a Poisson distribution to represent the duration of behavioral phases over consecutive steps.

Model parameters were estimated using maximum likelihood estimation (MLE), with confidence intervals obtained from profile likelihood analysis. Model fit was assessed by comparing the empirical distributions of step length and turning angles with the corresponding theoretical distributions based on MLE fitted parameters. Relative model performance was evaluated using the second order Akaike Information Criterion (AICc), which balances model fit and complexity while correcting for small sample sizes. Finally, the absolute goodness of fit of the best model was tested using a *G*‐test based on uniform pseudo residuals, with significance assessed at the 0.05 level. We processed and analyzed all data in R 4.4.2 (R Core Team 2024).

## 3 Results

**FIGURE 3:**
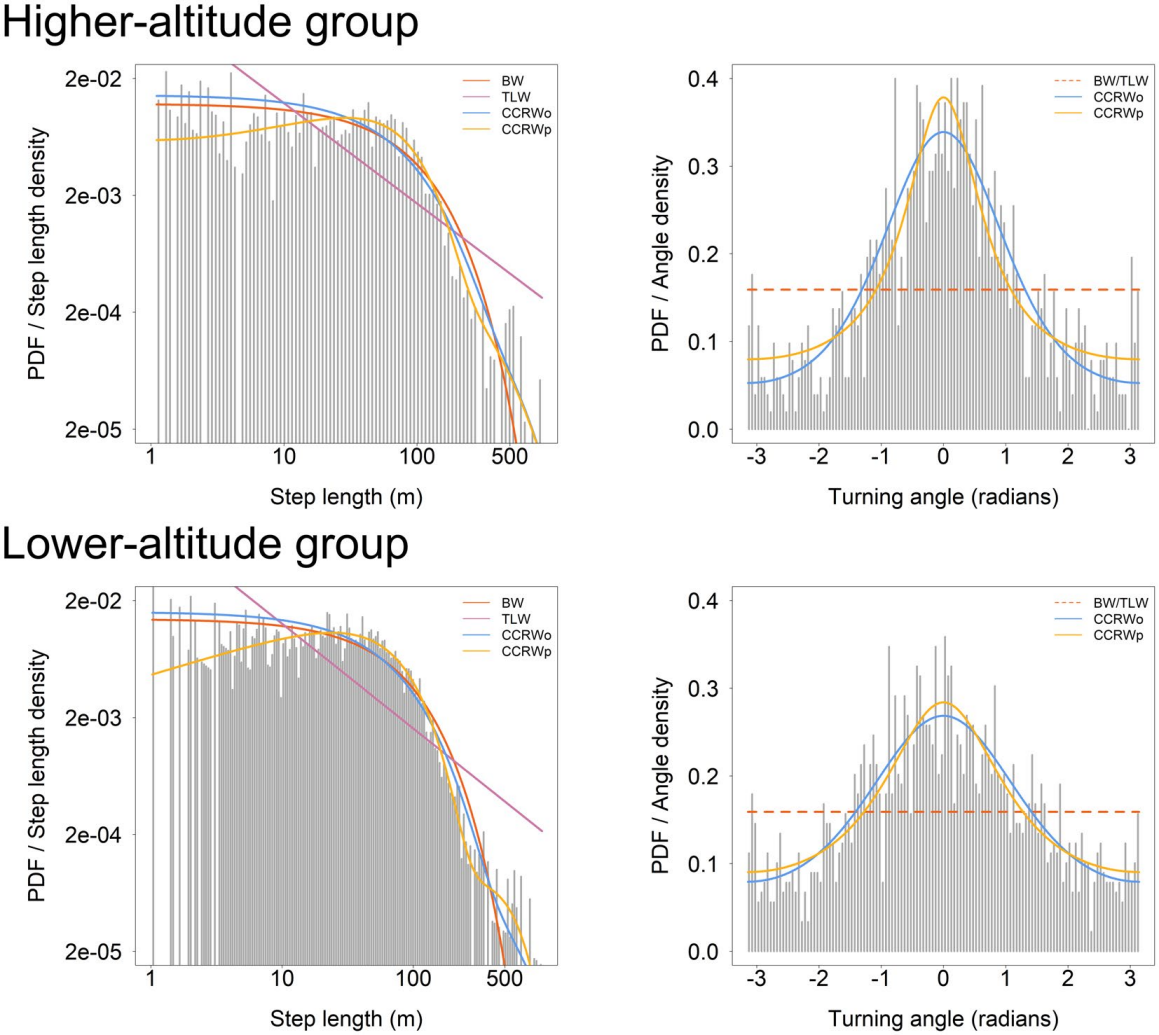
Step‐length and turning‐angle distributions for Higher‐altitude group and Lower‐altitude group. Panel (C) and panel (E) show the annual movement performance of each group. Gray bars represent the empirical distributions of step lengths or turning angles, and curves represent the theoretical probability density functions predicted by each model.

**FIGURE 4:**
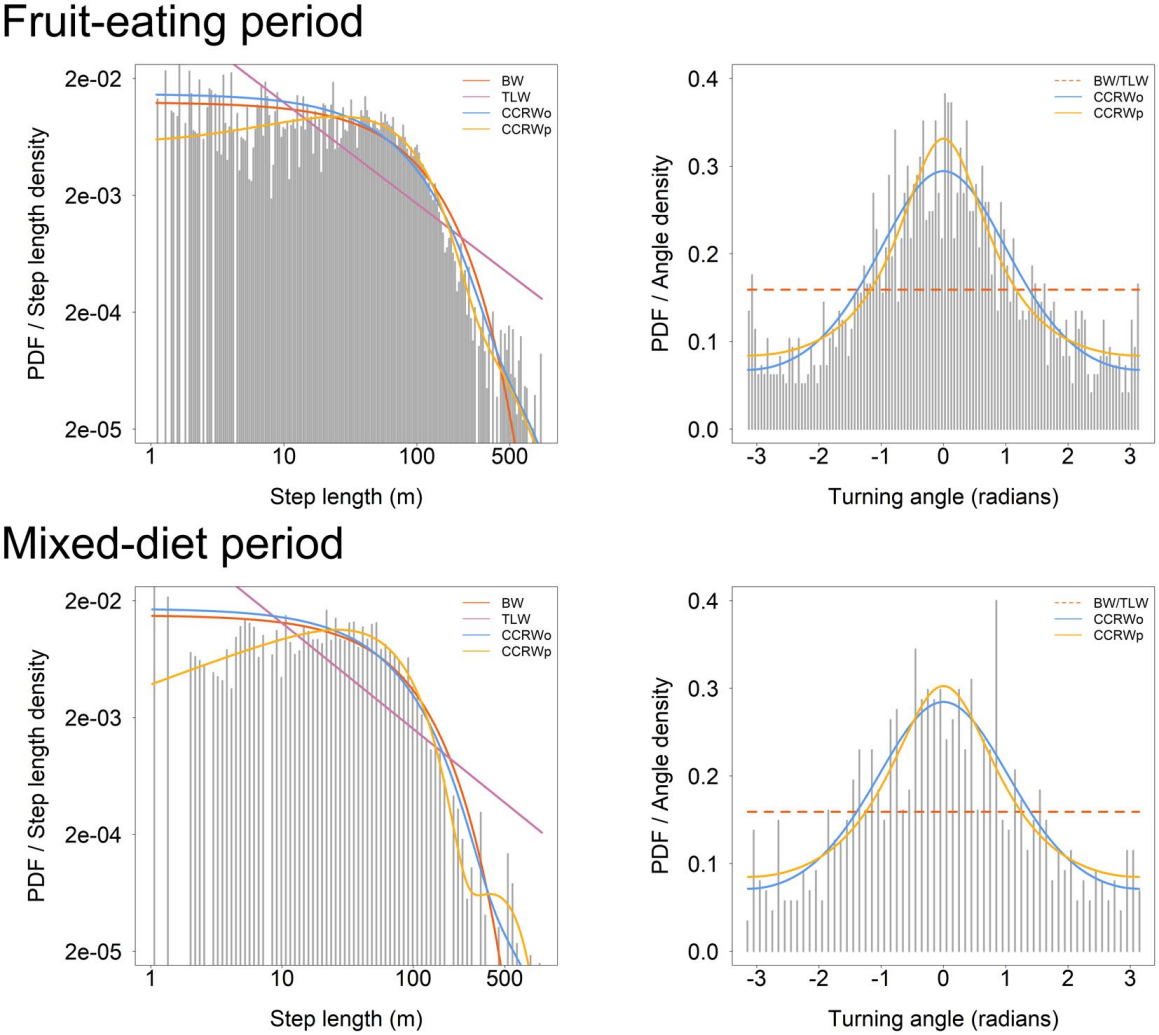
Step‐length and turning‐angle distributions during the Fruit‐eating and Mixed periods. Gray bars represent the empirical distributions of step lengths or turning angles, and curves represent the theoretical probability density functions predicted by each model.

Our model comparison identified CCRWp as the best‐supported model for the annual movement performance of the higher‐altitude and lower‐altitude groups, as well as for the movement performance of both groups during the fruit‐eating and mixed‐diet periods. This model showed the lowest AICc values, with Akaike weights exceeding 0.99 in all cases. In comparison, all other candidate models (BW, TLW, and CCRWo) exhibited higher AICc values, indicating little to no empirical support. Moreover, CCRWp provided a better fit to both step length and turning angle distributions than the other candidate models (Figures 3 and 4), indicating strong support for CCRWp relative to alternative models across these datasets. Based on the pseudo residual diagnostics, the CCRWp model showed no significant deviation for the annual movement performance of the higher‐altitude group (*p* = 0.11), whereas a significant deviation was detected for the annual movement performance of the lower‐altitude group (*p* = 0.02) (Table 1). For the CCRWp model fitted to the fruit‐eating period of the two groups (*p* = 0.76), and the mixed diet period of the groups (*p* = 0.24), no significant deviations were observed (Table 1).

**TABLE 1:** *p*‐values of model pseudo residuals. The goodness of fit of the optimal model for each gibbon group was assessed using pseudo residual diagnostics. HG indicated annual movement performance of Higher‐altitude group. LG indicated annual movement performance of Lower‐altitude group. FP indicated movement performance of both groups during the fruit‐eating period. MP indicated movement performance of both groups during the mixed‐diet period. PR‐*p* indicated overall pseudo residual *p*‐value. SL‐*p* indicated step length pseudo residual *p*‐value. TA‐*p* indicated turning angle pseudo residual *p*‐value.

Group	Model	PR‐*p*	SL‐*p*	TA‐*p*
HG	CCRWp	0.11	0.18	0.14
LG	CCRWp	0.02*	0.19	0.01**
FP	CCRWp	0.76	0.84	0.47
MP	CCRWp	0.24	0.32	0.20

*Note:* Asterisks indicate significant deviations of pseudo residuals from the expected distribution: * *p* < 0.05 and ** *p* < 0.01.

The movement patterns in the annual movement performance of both the higher‐altitude group and lower‐altitude group were dominated by the intensive search phase. However, the higher‐altitude group spent a greater proportion of time in the extensive search phase than the lower‐altitude group, with pE values of 0.22 and 0.04, respectively, while the mean step length during this phase was shorter in the higher‐altitude group than in the lower‐altitude group, with values of 152.22 and 388.68 m, respectively (Table 2). Gibbons relied primarily on the intensive search phase during both the fruiting and mixed‐diet periods. However, during the fruit‐eating period, gibbons spent a greater proportion of time in the extensive search phase than during the mixed‐diet period, with pE values of 0.16 and 0.03, respectively. The mean step length in this phase was also shorter during the fruit‐eating period than during the mixed‐diet period, with values of 163.72 m and 431.10 m, respectively (Table 2).

**TABLE 2:** States and movement parameters of the CCRWp model. HG indicated annual movement performance of Higher‐altitude group. LG indicated annual movement performance of Lower‐altitude group. pI and pE indicated the stationary proportions, referring to the proportion of time gibbons stay in the intensive search (I) or extensive search (E) state within a trajectory; sI and sE indicated the expected step lengths, representing the average step length in each state; rE indicated the turning angle concentration (mean resultant length), which was the concentration parameter of the Wrapped Cauchy distribution, with 0 indicating a circular uniform distribution of turning angles and values closer to 1 indicating a more concentrated turning angle distribution.

Group	Model	pI	pE	sI (m)	sE (m)	rE
HG	CCRWp	0.78	0.22	66.27	152.22	0.469
LG	CCRWp	0.96	0.04	61.23	388.68	0.428
FP	CCRWp	0.84	0.16	66.53	163.72	0.392
MP	CCRWp	0.97	0.03	57.72	431.10	0.317

## 4 Discussion

The movement patterns of Hainan gibbons were optimally described by a CCRW model, with the lowest AICc values, and the Akaike weights exceeding 0.99. This strategy consists of two distinct phases: an extensive search phase, characterized by long step lengths and highly directed movement (with turning angles following a Wrapped Cauchy distribution centered at 0), and an intensive search phase, dominated by short step lengths and sinuous, random movement (with turning angles approximating a circular uniform distribution). Such dual‐phase movement patterns are functionally comparable to variation in travel path sinuosity described in gibbons, where straighter paths are associated with between‐patch travel and more sinuous paths with within‐patch foraging (Brockelman 2009). Overall, the movement patterns of Hainan gibbons were dominated by the intensive search phase across both spatial and temporal scales, suggesting a general tendency toward localized foraging within resource patches. During the extensive search phase, gibbons maintained directional movement between fragmented or discontinuous food patches, which may facilitate efficient travel among spatially separated resources (Brockelman 2009; Hai et al. 2020). Because directed search increases energetic expenditure, whereas localized search may reduce movement costs while improving resource exploitation within patches, the foraging flexibility observed here may have important ecological and evolutionary significance in heterogeneous environments (Holyoak et al. 2008; Brockelman 2009; Light et al. 2026). The CCRWp model fitted to the annual movement performance of the lower‐altitude group did not pass the pseudo‐residual test, indicating limitations in fully capturing the complexity of the movement process. Nevertheless, it provided the best relative fit among the candidate models, as evidenced by the lowest AICc values across different groups and periods. Moreover, the movement characteristics captured by this model were, in overall terms, consistent with the movement patterns observed in the annual movement performance of the higher‐altitude group. Consequently, the annual movement performance of the lower‐altitude group was retained for comparative analyses, with interpretations restricted to relative differences in movement characteristics (Auger‐Méthé et al. 2016). Comparable movement‐based approaches have also been used in other gibbon studies to interpret responses to environmental variability and the distribution of important resources (Asensio et al. 2011; Hai et al. 2020; Zhang et al. 2025).

Hainan gibbons exhibit different movement patterns between the resource‐rich old‐growth forests and resource‐poor secondary forests (Table 2). Higher‐altitude group spent a greater proportion of time in the extensive search phase and exhibited stronger directional movement, but had shorter mean step lengths in this phase than lower‐altitude group. This difference may be related to variation in habitat quality between the two groups. Higher‐altitude group inhabits montane rainforests, where food resources are relatively abundant and canopy structure is more continuous and vertically complex, which may facilitate more efficient movement and flexible adjustment of movement strategies in response to seasonal resource variation (Liu et al. 2022, 2025; Wang et al. 2025; Du et al. 2020). In contrast, in lower quality lowland rainforests, canopy discontinuity, rugged terrain, and sparse food resources are likely to force individuals to trade off energy efficiency against safety, often reducing exploratory behavior and resulting in longer but less directionally persistent movements when traveling between resource patches (Du et al. 2020; Zhang et al. 2022; Liu et al. 2023; Wang et al. 2025). Habitat quality is not the only factor explaining the differences between the two groups. The two groups also differed in group size and adult composition, which may have influenced movement patterns through differences in group coordination, travel decision‐making, and responses to dispersed food resources (Brockelman 2009; Asensio et al. 2011). Previous studies have suggested that smaller gibbon groups may adjust travel direction more efficiently because movement decisions are shared among fewer individuals, and that group identity itself can explain substantial behavioral variation in gibbons (Asensio et al. 2011; Light et al. 2026). However, our study did not directly quantify canopy connectivity, seasonal resource variation, or the potential effects of group size and adult composition. Future studies incorporating these factors will be needed to better understand the mechanisms underlying the observed differences.

Across both the mixed‐diet and fruit‐eating periods, Hainan gibbons exhibited a consistent dual phase, flexible search strategy. However, the proportion of time allocated to the extensive search phase was higher during the fruit‐eating period than during the mixed‐diet period (Table 2). This difference is likely driven by seasonal variation in food availability, as fruits represent high‐energy but patchily distributed resources, whereas leaves function as lower‐energy fallback foods (Milton 1980; He et al. 2023; Zhong et al. 2025). Hainan gibbons adjusted the relative allocation of time between extensive and intensive search phases across periods of varying resource availability. During periods of lower fruit availability, they spent more time in localized search, whereas during periods of higher fruit availability, they allocated more time to extensive search. This pattern is consistent with previous studies showing that gibbons reduce travel and increase energy‐conserving behaviors when resources are scarce, but increase movement and ranging effort when resources are more abundant (Light et al. 2026; Hai et al. 2020). This behavioral flexibility is consistent with optimal foraging theory, which predicts that animals should adjust their foraging strategies to maximize net energy gain by balancing energy intake against the costs of movement and foraging (Charnov 1976; Hopkins 2016; Nagy‐Reis and Setz 2017; Xue et al. 2023; Zhang et al. 2024). Similar behavioral responses have also been observed in other primates under fluctuating resource conditions. For example, ring‐tailed lemurs ( *Lemur catta* ) and Bale monkeys ( *Chlorocebus djamdjamensis* ) inhabiting fragmented environments exhibit energy‐minimizing strategies characterized by reduced travel distances and increased residence time within patches (Mekonnen et al. 2017; Hending et al. 2024). In gibbons specifically, activity patterns and ranging behavior have been shown to vary in response to habitat quality and resource availability. When food resources are scarce or unevenly distributed, gibbons tend to reduce travel distance, increase resting time or localized foraging, and concentrate their activities within available food patches; when resources are more abundant, they increase movement and ranging effort to exploit dispersed food resources (Light et al. 2026; Hai et al. 2020; Saralamba et al. 2022).

## 5 Conclusion

Gibbons adopt a dual mode behavioral strategy, exhibiting flexible movement behavior and potential energy optimizing characteristics across both temporal (fruit‐eating and mixed‐diet periods) and spatial scales (higher‐altitude group inhabits resource rich old‐growth tropical forests; lower‐altitude group inhabits resource poor secondary tropical forests). Such foraging flexibility reflects their high adaptability to habitat conversion, suggesting that secondary forests play an important role in sustaining gibbon populations and merit further protection and restoration. Furthermore, since gibbon movement is tightly linked to food phenology, long term monitoring of key food plant species is essential. Restoration of these critical food resources will bolster the ecological resilience and long‐term stability of Hainan gibbon populations. This study included only two groups. Given the extremely small population size of Hainan gibbons, this small sample size limits the extent to which our findings can be generalized to the species as a whole. The two study groups occupied different habitat conditions and therefore provide valuable insights into how movement patterns may vary across different ecological contexts. However, other groups may differ in habitat use, group composition, or ranging behavior, and may not show the same patterns observed in this study. Therefore, future studies including additional groups and longer‐term comparisons are needed to assess the broader generality of these movement patterns.

## Author Contributions

**Wei Yang:** data curation (equal), formal analysis (lead), investigation (equal), visualization (lead), writing – original draft (lead), writing – review and editing (equal). **Dexu Zhang:** data curation (equal), investigation (equal). **Shuai Liu:** data curation (equal), investigation (equal). **Kening Lu:** data curation (equal), investigation (equal). **Chuyue Yu:** data curation (equal). **Changyuan Su:** data curation (equal), investigation (equal). **Yuan Chen:** conceptualization (equal), funding acquisition (lead), project administration (lead), supervision (equal), writing – review and editing (equal). **Wenxing Long:** conceptualization (equal), funding acquisition (lead), project administration (lead), supervision (lead), writing – review and editing (equal).

## Funding

This work was supported by National Natural Science Foundation of China, U22A20503, Project of the Hainan University Collaborative Innovation Center, XTCX2022STC24, Innovational Fund for Scientific and Technological Talent of Hainan Province, KJRC2023C13, Hainan Provincial Natural Science Foundation of China, 324QN210.

## Ethics Statement

The study adhered to the American Society of Primatologists' principles for the ethical treatment of primates. Fieldwork permits were obtained from the relevant local authorities, including the Bawangling National Nature Reserve Administration.

## Conflicts of Interest

The authors declare no conflicts of interest.

## Data Availability

The data that support the findings of this study are openly available in Figshare: https://figshare.com/articles/dataset/30921101.
